# Uclacyanin MtUC1 Is Involved in the Regulation of Nodule Senescence in *Medicago truncatula*

**DOI:** 10.1111/mpp.70171

**Published:** 2025-11-12

**Authors:** Li Wang, Mengdi Zhang, Wenjun Tan, Zhaoyang Yang, Shicheng Zhao, Mengzhen Jia, Gehong Wei, Minxia Chou

**Affiliations:** ^1^ State Key Laboratory for Crop Stress Resistance and High‐Efficiency Production, Shaanxi Key Laboratory of Agricultural and Environmental Microbiology, College of Life Sciences Northwest A&F University Yangling Shaanxi China; ^2^ College of Life Sciences Anqing Normal University Anqing Anhui China; ^3^ Civil and Environmental Engineering Harbin Institute of Technology Shenzhen China

**Keywords:** bacteroid survival, early senescence, *Medicago truncatula*, nodule development, uclacyanin

## Abstract

Phytocyanins (PCs) are ancient plant‐specific blue copper proteins that play an important role in plant growth and development, and stress tolerance. In this study, the role of MtUC1, a member of the uclacyanin subfamily of the PC family, was analysed in the nodule symbiosis of *Medicago truncatula* . *MtUC1* was mainly expressed in the nodule interzone and strongly induced in the later nodule developmental stage. RNA interference (RNAi) and mutation of *MtUC1* led to reduced root nodule formation and degeneration of bacteroids within nodules. Cysteine protease activity in the *MtUC1‐RNAi* inoculated roots and *uc1* mutant nodules was significantly increased, the leghaemoglobin content and the expression of nitrogen‐fixing enzyme genes in the *uc1* mutant nodules were significantly reduced, and the nodule cells showed signs of senescence, suggesting that MtUC1 expression is required to avert nodule senescence. Transcriptomic analysis indicated that many symbiotic genes were significantly downregulated, and the senescence/defence‐related genes were significantly upregulated in roots 7 days post‐inoculation (dpi) and in the nodules of the *uc1* mutant at 28 dpi. Yeast two‐hybrid and bimolecular fluorescence complementation experiments showed that MtUC1 interacted with MtBI‐1 (Bax‐Inhibitor 1). Both MtUC1 and MtBI‐1 were localised and co‐localised to the endoplasmic reticulum and plasma membrane. In addition, *MtBI‐1* also showed a significantly high expression level in the mature nodules. In summary, MtUC1 may prevent the premature aging of root nodules by interacting with MtBI‐1.

## Graphical abstract

MtUC1, a member of the uclacyanin subfamily of the phytocyanin family, may maintain the survival of bacteroids in the nodule cells and prevent nodules from premature aging by interacting with MtBI‐1.

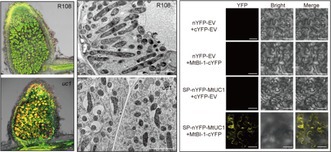

## 1 Introduction

Symbiotic nitrogen fixation between legumes and rhizobia is a complex biological process strictly regulated by the host plant. The roots of host legumes secrete flavonoids and other chemo‐attractants into the rhizosphere to recruit rhizobia when environmental nitrogen is deficient (Compton and Scharf 2021). After sensing and binding to specific flavonoids secreted by host plants, the NodD protein in rhizobia is activated to induce the synthesis of nodulation factors (NFs). NFs are recognised by the receptor complexes on the surface of the host root cell membrane and give rise to the root hair curling to entrap the rhizobia in an infection chamber (Bozsoki et al. 2020; Moling et al. 2014). Subsequently, the infection chamber changes from radial to tubular growth inwardly associated with remodelling of the surrounding walls and a local invagination of the plasma membrane to form an infection thread (IT), in which the rhizobia divide and multiply (Fournier et al. 2015; Gage 2004). Simultaneously, the partial cells of the pericycle, endodermis and cortical resume their division activity and divide to form the nodule primordia. As the ITs extend forward through the cortex cells to reach the nodule primordia, the rhizobia are released into the nodule primordial cells through unwalled droplets and terminally differentiate into bacteroids, which are encapsulated by the symbiosome/peribacteroid membrane derived from the host, namely symbiosomes (Oldroyd and Downie 2004).

Generally, nodules are classified into two types: indeterminate and determinate nodules. Indeterminate nodules, formed in legumes such as *Medicago truncatula* , *Vicia sativa* and *Trifolium repens* , are characterised by distinct internal zones: a persistent apical meristem zone (zone I, ZI), an infection zone (zone II, ZII) where rhizobia are delivered to the nodule primordia from the ITs and subsequently divide, an interzone (IZ) where rhizobia differentiate into bacteroids, a nitrogen fixation zone (zone III, ZIII) where bacteroids within the symbiosomes fix nitrogen via nitrogenase activity, and a senescence zone (zone IV, ZIV) where both host cells and bacteroids undergo degradation (Timmers et al. 2000; Vasse et al. 1990). Determinate nodules, such as those formed on the roots of *Glycine max* and *Lotus japonicus*, are characterised by the absence of a persistent meristem and distinct internal zones when the nodules mature (Puppo et al. 2005). In addition, there is a lack of terminal bacteroid differentiation in the determinate nodules.

Phytocyanin (PC) family members are ancient plant‐specific type I blue copper proteins that can bind a single copper atom and function as electron transporters in various biological cells (Giri et al. 2004). The PC family can be divided into four subfamilies: plantacyanin (PLC), uclacyanin (UC), stellaracyanin (SC) and early nodulin‐like protein (ENODL) (Sun et al. 2019; Wang, Zhang, et al. 2023). Among them, uclacyanin is a plant‐specific cell wall protein consisting of a copper‐binding site and a domain similar to cell wall structural proteins and is involved in fibre formation and/or tissue lignification (Nersissian et al. 1998). Previous studies show that *VviUCC1* (uclacyanin 1‐like) encodes a uclacyanin protein involved in the lignification of the inflorescence rachis in *Vitis vinifera* . This lignification occurs during the elongation and thickening processes of the inflorescence axis and provides the support for the fundamental structures required to bear the weight of grapes (Tello et al. 2020). Loss‐of‐function of *AtUCC1* (uclacyanin1) and *AtUCC2* in *Arabidopsis thaliana* reduces lignification of the central Casparian strip nanodomain and increases endodermal permeability, ultimately leading to a loss of mineral nutrient homeostasis (Reyt et al. 2020). Interestingly, some uclacyanins are also the regulatory targets of microRNAs (miRNAs) in plants (Gao et al. 2022; Song et al. 2018). *Oryza sativa* OsmiR408 regulates rice yield by downregulating its downstream target, *OsUCL8* (uclacyanin) (Zhang et al. 2017, 2018). In addition, OsmiR528 regulates pollen intine formation and flavonoid metabolism by directly targeting OsUCL23 (Zhang et al. 2020). Uclacyanins also play a role in plant response and defence against abiotic stresses such as drought, salinity, high temperatures and cold (Cao et al. 2015; Ma et al. 2011). Studies have shown that AtUC5 may mediate plant responses to ozone stress by regulating the generation or signalling of reactive oxygen species; overexpression of *AtUC5*, *AtUC6* and *AtSC3* confers ozone tolerance in *Arabidopsis*, thereby protecting plants from oxidative stress (Saji et al. 2022).

Our laboratory previously characterised and analysed members of PC gene family in *M. truncatula* and found that silencing the *MtENODL27* and *MtENODL28* genes using RNA interference (RNAi) resulted in impaired rhizobial infection, a reduced number of nodules and diminished nitrogen fixation capacity (Sun et al. 2019). On this basis, the relative expression levels of PC family members in the nodule were analysed using the published Symbimics database (https://iant.toulouse.inra.fr/symbimics/) (Roux et al. 2014), and *MtUC1* was identified with the highest relative expression in the interzone of nodules. In this study, the expression patterns of *MtUC1* were investigated, and RNAi and *Tnt1* insertion mutants of *MtUC1* were used to examine its specific role in symbiotic nodulation. Yeast two‐hybrid (Y2H) assays and bimolecular fluorescence complementation (BiFC) were used to identify the interaction between MtUC1 and MtBI‐1. Our findings demonstrate that MtUC1 may play a positive role in the symbiotic nitrogen fixation of *M. truncatula* by interacting with MtBI‐1.

## 2 Results

### 2.1 *MtUC1* Was Highly Expressed in the Interzone of Mature Nodules

**FIGURE 1:**
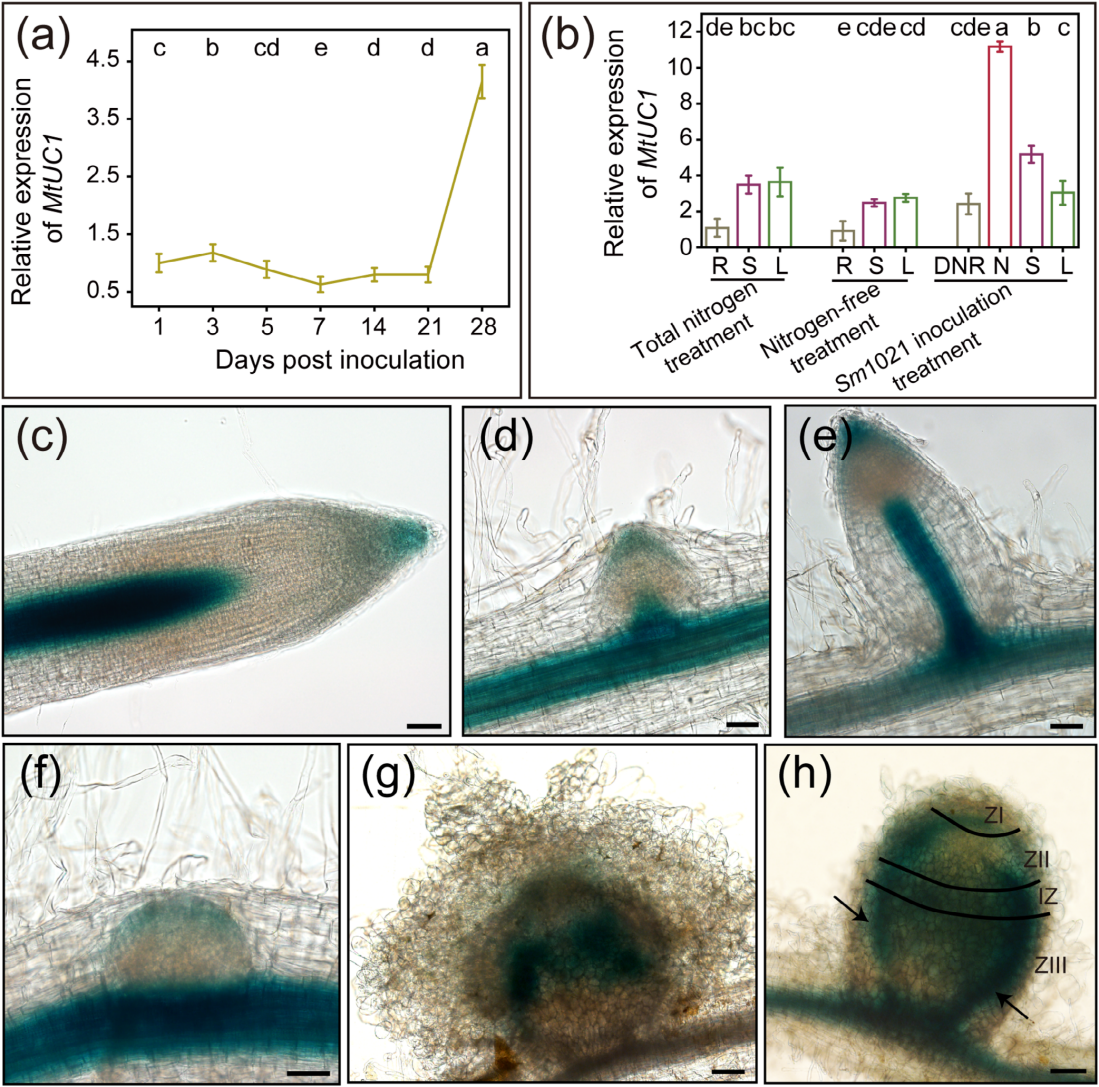
Expression pattern of *MtUC1*. (a, b) Spatial and temporal expression patterns of *MtUC1*. Roots were obtained at 1, 3, 5, 7, 14, 21 and 28 days post‐inoculation (dpi) (a). The roots (R), stems (S), leaves (L), denodulated roots (DNR) and nodules (N) were harvested at 28 dpi under the three treatments of total nutrition (with nitrogen), nitrogen deficiency and inoculation with *Sinorhizobium meliloti* 1021 (b). *MtActin* and *MtEF* were used as reference genes. Values show mean ± standard deviation for a total of three biological repeats, with three technical repeats included in each biological repeat. The same lowercase letters show no significant difference, and different lowercase letters show a significant difference (Tukey's test, *p* < 0.05). (c–e) Under total nutrition treatment, the *pMtUC1:GUS* reporter gene was expressed in roots (c, 14 days), lateral root primordia (d, 14 days), and lateral roots (e, 14 days). (f–h) Under inoculation with *S. meliloti* 1021, expression patterns of *pMtUC1:GUS* reporter in nodule primordia (f, 7 dpi), young nodules (g, 14 dpi) and mature nodules (h, 28 dpi). Images are representative of at least 10 independent transgenic plants. Arrows point to vascular bundles. IZ, interzone; ZI, meristem zone; ZII, infection zone; ZIII, nitrogen fixation zone. Scale bars, 50 μm (c–g); 100 μm (h).

To determine the potential functions of PC family members during symbiotic nitrogen fixation, the gene expression profiles of *MtPC*s in different zones of nodule were analysed using the Symbimics database. Among the 82 identified MtPCs, only 51 had corresponding expression data in the Symbimics database. The 51 *MtPC*s showed distinct expression patterns across different zones of the nodules and could be broadly categorised into four types: (1) meristem zone‐enriched expression type, exemplified by *MtSC6* (*Medtr4g066110*), *MtSC7* (*Medtr4g067200*) and others; (2) infection zone‐enriched expression type, represented by *MtENODL29* (*Medtr5g087970*) and similar genes; (3) interzone‐enriched expression type, including *MtUC1* (*Medtr1g112700*) and related genes; (4) nitrogen fixation zone‐enriched expression type, such as *MtPLC1* (*Medtr3g089005*) (Figure S1 and Table S1). In this study, we focused on the gene *MtUC1*, which exhibited the highest expression level in the interzone compared to other zones and showed a higher expression level in the interzone relative to other *MtPC*s (Figure S1, Table S1). Subsequently, to explore the expression pattern of *MtUC1* during nodulation, the expression level of *MtUC1* was analysed using reverse transcription‐quantitative PCR (RT‐qPCR). The results showed that *MtUC1* was strongly induced in the late stage of nodule development, in contrast to its relatively low expression during the early and middle stages (Figure 1a). The analysis of *MtUC1* expression levels in different tissues under different treatments showed that *MtUC1* was expressed in all tissues examined. Compared with the nitrogen‐free treatment, the expression of *MtUC1* in the underground parts and stems was strongly induced by the rhizobium inoculation, with the highest expression in nodules (Figure 1b).

To further investigate the expression pattern of *MtUC1* in roots and nodules, transgenic plants with the *MtUC1* promoter fused to β‐glucuronidase (GUS) were obtained via *Agrobacterium rhizogenes* ‐mediated hairy root transformation of *M. truncatula* . GUS staining was performed on the roots of transgenic plants treated with full nutrition (nitrogen‐containing), and the results showed that *MtUC1* was expressed in the tips and stele of primary and lateral roots (Figure 1c–e). Histochemical analysis of the roots of transgenic plants at 7, 14 and 28 days post‐inoculation (dpi) revealed that GUS staining was mainly detected in the apical part of the nodule primordia (Figure 1f), the middle zone of young nodules (Figure 1g) and the interzone and peripheral vascular bundles of mature nodules (Figure 1h). These results were largely consistent with the data in the Symbimics database (Figure S1, Table S1).

### 2.2 Silencing and Mutation of *MtUC1* Accelerated the Senescence of Nodules

**FIGURE 2:**
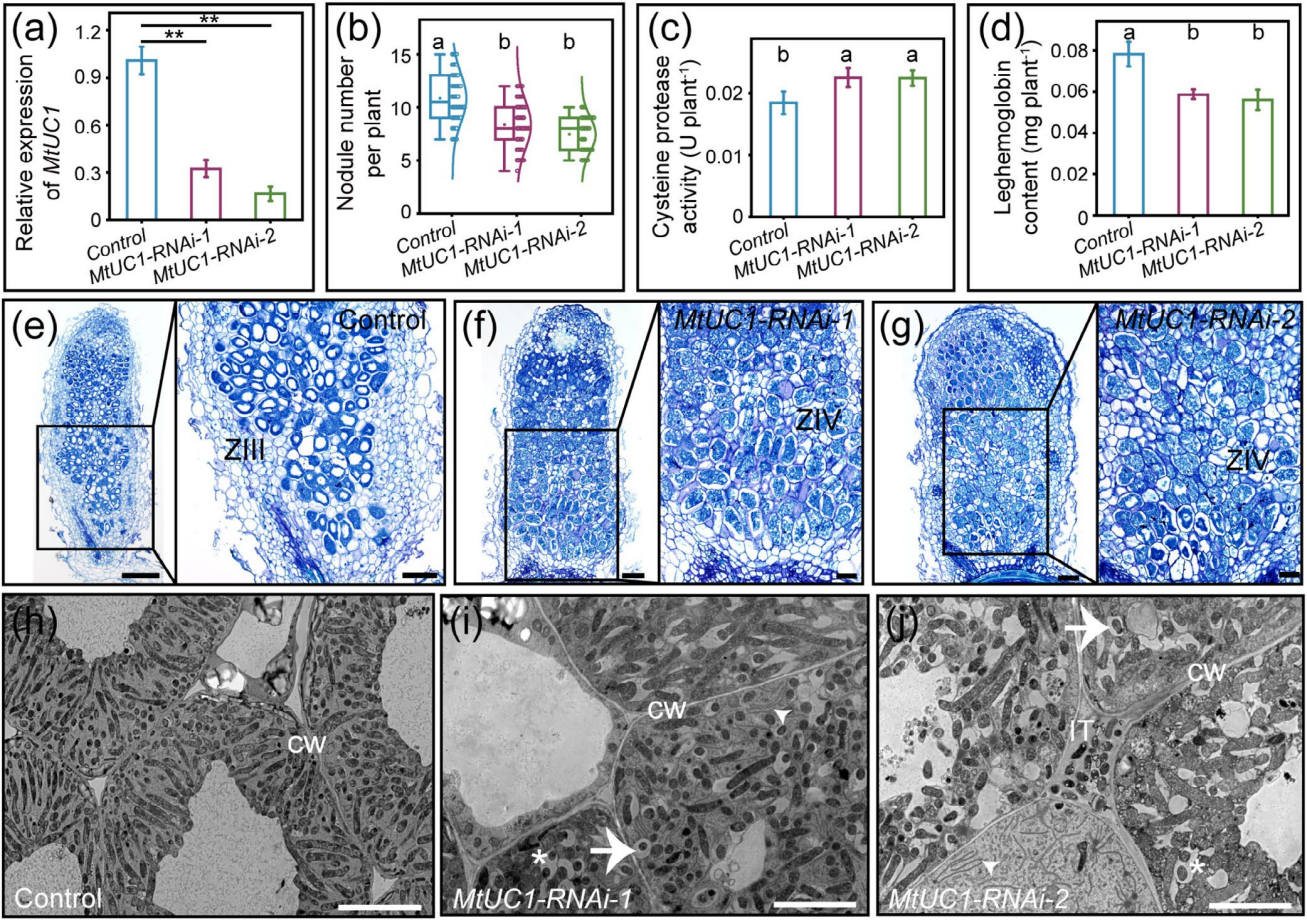
Nodulation characterisation of *MtUC1‐RNAi* plants. (a–d) Relative expression level of *MtUC1* (a), nodule number (b), cysteine protease activity (c) and leghaemoglobin content (d) in *MtUC1‐RNAi* inoculated roots (28 days post‐inoculation [dpi]). *MtActin* and *MtEF* were used as reference genes. Values are derived from three biological replicates and three technical replicates, and the error bar is the standard deviation. Asterisks indicate that the expression of *MtUC1* is significantly different (Student's *t* test, **p < 0.01). The same lowercase letters show no significant difference, and different lowercase letters show a significant difference (Tukey's test, *p* < 0.05). (e–g) Paraffin sections of control (e) and *MtUC1‐RNAi* (f, g) nodules (28 dpi). The insets (right) are enlargements in the black boxes (left). ZIII, nitrogen fixation zone; ZIV, senescence zone. Scale bars, 200 μm (e–g, left); 100 μm (e–g, right). (h–j) Transmission electron microscope images of the nitrogen‐fixation zone of control (h) and *MtUC1‐RNAi* (i, j) nodules (28 dpi). CW, cell wall; IT, infection thread. Arrowhead, arrow and asterisk indicate peribacteroid space, endoplasmic reticulum and fusion between symbiosomes, respectively. Scale bars, 10 μm.

To investigate the role of MtUC1 in symbiotic nodules, two independent RNAi constructs were introduced into *M. truncatula* roots via *A. rhizogenes* ‐mediated transformation to specifically silence the expression of *MtUC1* (Figure S2). Transgenic plants were observed using fluorescence microscopy to select the *MtUC1‐RNAi* positive plants (Figure S3). Next, the transcript levels of *MtUC1* in the *MtUC1‐RNAi* roots were analysed by RT‐qPCR, and the results showed that expression of *MtUC1* was knocked down (Figure 2a).

Compared with the control, the nodule number of *MtUC1‐RNAi* plants was significantly reduced (Figure 2b). Cysteine protease (CP) activity was significantly increased (Figure 2c), and leghaemoglobin (Lb) content was significantly reduced in the inoculated roots of *MtUC1‐RNAi* compared with that in the control (Figure 2d). Next, microscopic and ultramicroscopic structural analyses were performed on the nodules at 28 dpi. The observation of paraffin sections revealed that the *MtUC1‐RNAi* nodules showed signs of aging, such as the disappearance of large central vacuoles in the infected cells, while in the control nodule cells, a large vacuole was observed that returned in volume to pre‐infection level (Figure 2e–g), which indicated the completion of microsymbiont terminational differentiation and the maturation of nitrogen‐fixing symbiosomes (Gavrin et al. 2014). The results of ultrathin section analysis further showed that in the infected cells of *MtUC1‐RNAi* nodules, a large amount of endoplasmic reticulum (ER) appeared in the nodule cells, the bacteroid space was significantly enlarged, the fusion occurred between some symbiosomes, and individual symbiosomes have been degraded, while the bacteroids were well differentiated in the infected cells of control nodules (Figure 2h–j).

**FIGURE 3:**
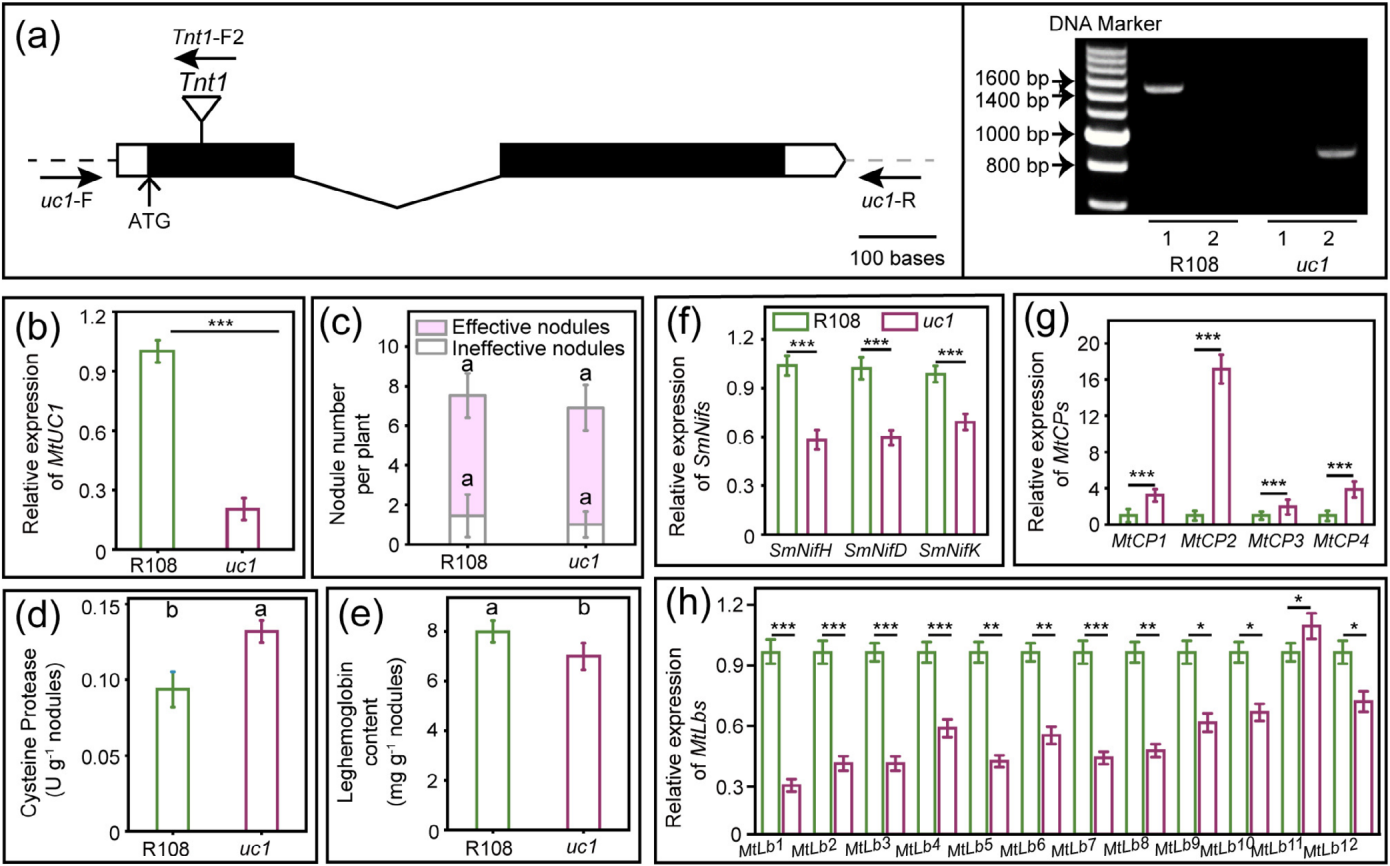
Nodulation characterisation of *uc1* mutants. Insertion sites of *Tnt1* in the *uc1* mutant (a, left). The black solid boxes, black broken line, black dotted line and grey dotted line represent the exons, intron, partial promoter and partial terminator of *MtUC1*, respectively. Scale bar of the *MtUC1* gene, 100 bases. Detection of a *uc1* homozygous mutant line with a *Tnt1* insertion (a, right). Band 1 was amplified using the forward primer *uc1*‐F and the reverse primer *uc1‐*R of the *MtUC1* gene, and band 2 was amplified using the forward primer *uc1*‐F of the *MtUC1* gene and the forward primer *Tnt1*‐F2 of *Tnt1*. (b–h) Relative expression levels of *MtUC1* (b), *SmNif*s (f), *MtCP*s (g) and *MtLb*s (h), and nodule number (c), cysteine protease activity (d) and leghaemoglobin content (e) in wild‐type R108 and *uc1* mutant nodules (28 days post‐inoculation). *Sm16SrRNA* and *SmuppS* were used as reference genes for *SmNif*s, and *MtActin* and *MtEF* were used as reference genes for *MtUC1*, *MtCP*s and *MtLb*s. Values are derived from three biological replicates and three technical replicates, and the error bar is the standard deviation. Asterisks indicate that the expression of the gene is significantly different (Student's *t* test, *p < 0.05, **p < 0.01, ***p < 0.001). The same lowercase letters show no significant difference, and different lowercase letters show a significant difference (Tukey's test, *p* < 0.05).

Nodulation analysis of *MtUC1‐RNAi* plants showed that MtUC1 impacted the senescence of nodules. Next, *uc1* mutant lines were screened, and their growth and nodulation phenotypes were analysed to confirm the previous results. Genotyping and PCR assays showed that *Tnt1* was inserted in reverse in the *uc1* mutant line (NF10257) (Figure 3a). RT‐qPCR results showed that the expression of *MtUC1* was significantly downregulated in the *uc1* mutant as compared with that in the wild‐type R108 (Figure 3b).

**FIGURE 4:**
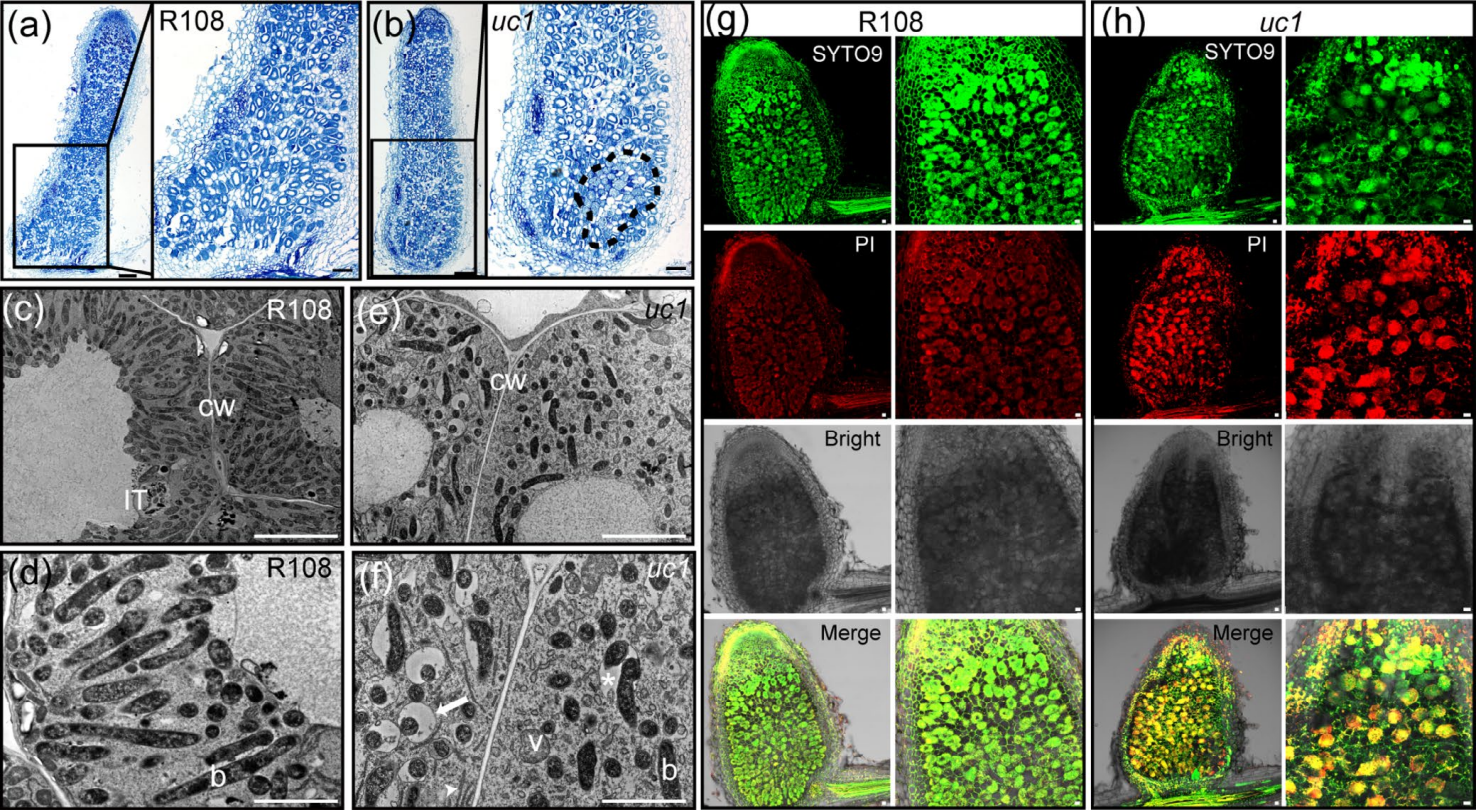
Nodule sections of wild‐type R108 and *uc1* mutants. (a, b) Paraffin sections of wild‐type R108 (a) and *uc1* mutant (b) nodules (28 days post‐inoculation [dpi]). Infected cells with signs of senescence within the nitrogen fixation zone are shown in the dotted area (b, right). The insets (right) are enlargements in the black boxes (left). Scale bars, 200 μm (a, b, left); 100 μm (a, b, right). (c–f) Transmission electron microscope images of the nitrogen‐fixation zone of wild‐type R108 (c, d) and *uc1* mutant (e, f) nodules (28 dpi). b, bacteroid; CW, cell wall; IT, infection thread; v, vesicle. Arrowhead, arrow and asterisk indicate peribacteroid space, endoplasmic reticulum and fusion between symbiosomes, respectively. Scale bars, 10 μm (c, e); 2 μm (d, f). (g, h) Live/dead staining of wild‐type R108 (g) and *uc1* mutant (h) nodule sections (14 dpi) with SYTO9 and propidium iodide (PI). Scale bars, 40 μm.

There was no significant difference in the nodule number between the *uc1* mutant and wild‐type R108 (Figure 3c). However, compared with that in the wild‐type nodules, the CP activity and the expression of four *MtCP* genes were significantly increased in the *uc1* mutant nodules; the Lb content and the expression of all 11 *MtLb* genes except *MtLb11* were significantly reduced in the *uc1* mutant nodules. Furthermore, the expression of nitrogen‐fixing enzyme genes was significantly downregulated in the *uc1* mutant nodules (Figure 3d–h). The analyses of paraffin sections and ultrathin sections of the 28 dpi nodules displayed that although the senescence phenotype of the *uc1* mutant nodules was milder than that of the *MtUC1‐RNAi* nodules, both showed a reduction in vacuoles compared with the control (Figures 2e–g and 4a,b). Compared with that in the wild‐type nodules (Figure 4c,d), the bacteroid space increased and symbiosome fusion occurred in the *uc1* mutant nodules. Moreover, a large number of vesicles and ER appeared in the infected cells of the nitrogen‐fixing zone in the *uc1* mutant nodules (Figure 4e,f), indicating that bacteroids were being degraded.

In addition, the live/dead cell staining analysis of young nodules (14 dpi) showed that the infected cells of *uc1* mutant nodules contained more dead bacteroids (stained red) than those of wild‐type nodules (Figure 4g,h), implying that MtUC1 may contribute to sustain bacteroid viability during symbiosis.

### 2.3 Symbiotic Genes Were Significantly Downregulated and the Senescence/Defence‐Related Genes Were Highly Induced During the Nodulation of the *uc1* Mutant

**FIGURE 5:**
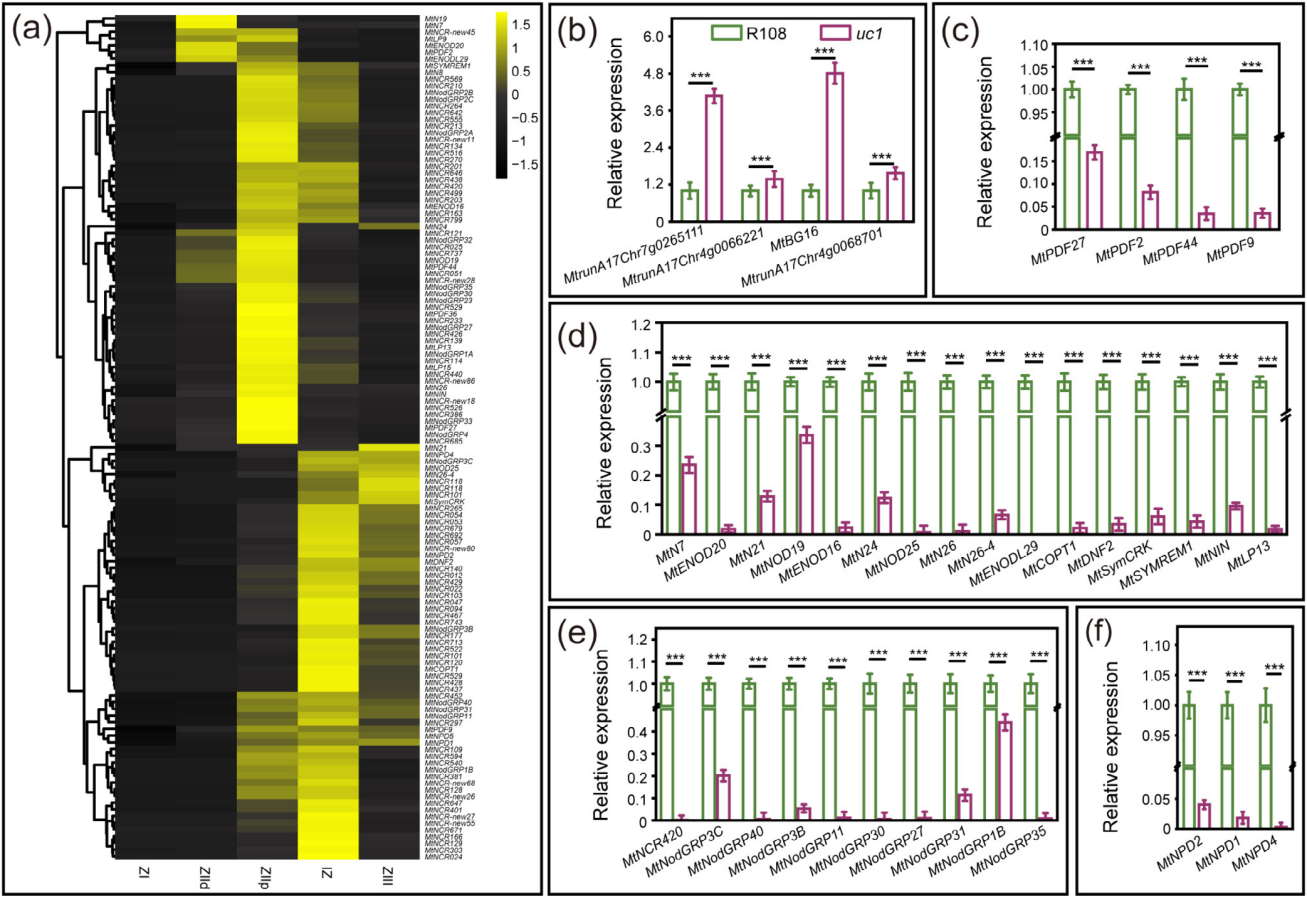
Analysis of differentially expressed genes in the inoculated roots of *uc1* mutant. (a) Relative expression levels of the significantly downregulated genes in different regions of nodules. Data shown in the heat map were obtained from the Symbimics database. IZ, interzone; ZI, meristem zone; ZIId, distal infection zone; ZIIp, proximal infection zone; ZIII, nitrogen fixation zone. Black shows low expression, and yellow shows high expression. (b–f) Reverse transcription‐quantitative PCR validation of the significantly upregulated genes associated with cell wall remodelling and stress response and adaptation (b) and the significantly downregulated genes (c–f) in the inoculated roots of *uc1* mutant (7 dpi), including the symbiotic nitrogen fixation‐related genes (c, d), NCR and NodGRP genes (e) and genes encoding nodule‐specific PLAT domain proteins (f). *MtActin* and *MtEF* were used as reference genes. Values are derived from three biological replicates and three technical replicates, and the error bar is the standard deviation. Asterisks indicate that the expression of the gene is significantly different (Student's *t* test, ***p < 0.001).

To investigate the impact of MtUC1 on the transcript levels of genes related to the early symbiotic process, RNA‐seq analysis was performed on the inoculated roots of wild‐type R108 and *uc1* mutant at 7 dpi. The results showed that compared to wild‐type R108, 640 genes were significantly downregulated in the inoculated roots of *uc1* mutant at 7 dpi. Among these significantly downregulated genes, there were 78 genes encoding nodule‐specific cysteine‐rich (NCR) peptide, 17 genes encoding nodule‐specific glycine‐rich proteins (NodGRPs), 5 genes encoding symbiotic defensin/Mtnodulin/Plant Defensin, 4 genes encoding nodule‐specific polycystin‐1, lipoxygenase, α‐toxin (PLAT) domain proteins and 20 other symbiotic genes, such as *MtNIN* (Liu et al. 2021), *MtSYMREM1*/*MtREM2*.2 (Lefebvre et al. 2010), *MtSymCRK* (Berrabah et al. 2014), *MtDNF2* (Bourcy et al. 2013), *MtENOD16* and *MtENOD20* (Greene et al. 1998) (Table S2). Cluster analysis based on the Symbimic database showed that most of these genes were highly expressed in the proximal infection zone and interzone of wild nodules (Figure 5a). Furthermore, a total of 247 genes were significantly upregulated in the 7‐dpi inoculated roots of *uc1* mutant, including 51 genes involved in the transcriptional regulation process and 9 genes associated with cell wall remodelling and stress response and adaptation (Table S3). The results of transcriptome sequencing were also further verified using RT‐qPCR (Figure 5b–f).

**FIGURE 6:**
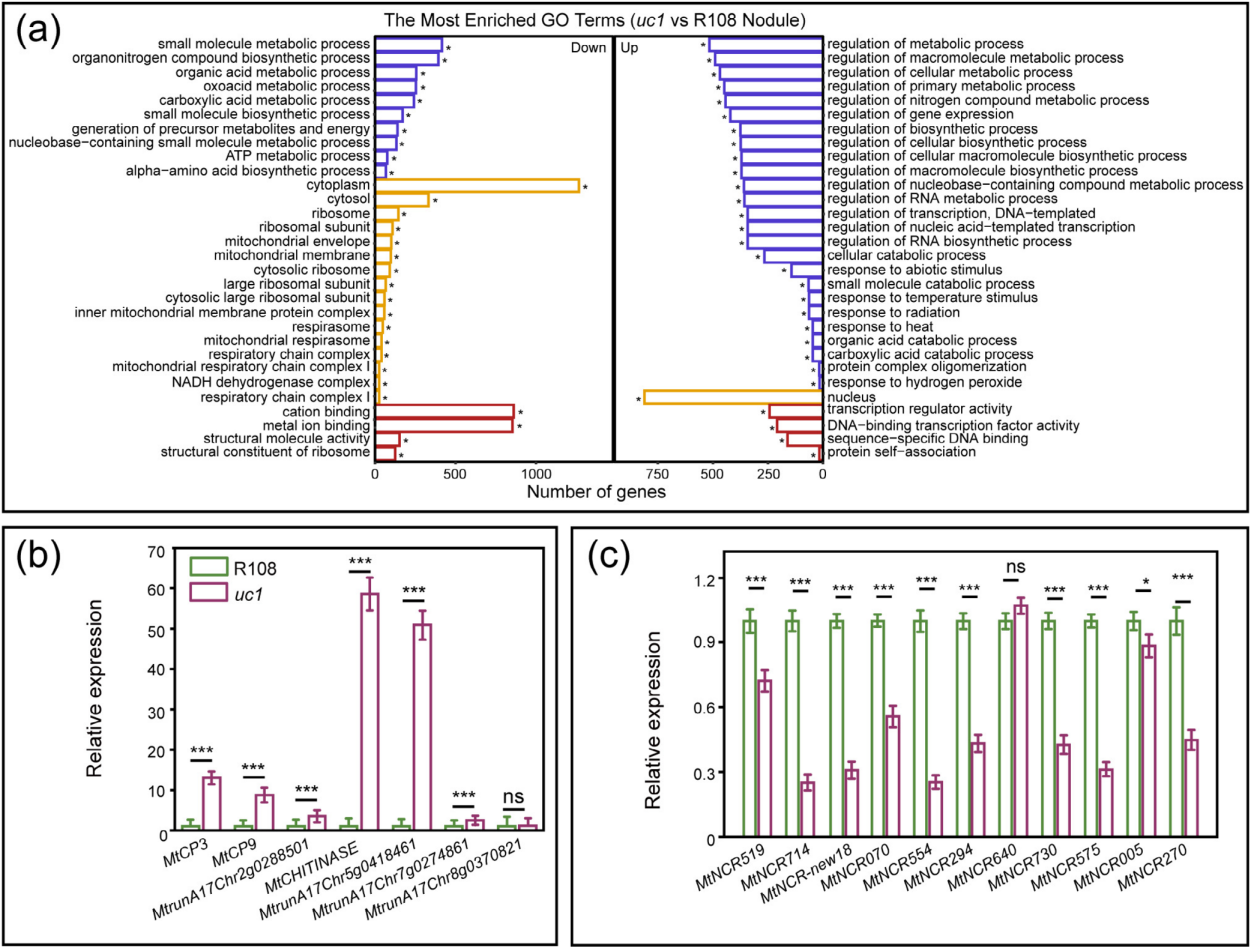
Analysis of differentially expressed genes in the nodules of *uc1* mutant. (a) GO enrichment histogram of upregulated (left) and downregulated (right) genes in the nodules of *uc1* mutant (28 days post‐inoculation [dpi]). (b, c) Reverse transcription‐quantitative PCR validation of the significantly upregulated genes related to senescence and defence responses (b) and the significantly downregulated genes encoding NCR peptides (c) in the nodules of *uc1* mutant (28 dpi). *MtActin* and *MtEF* were used as reference genes. Values are derived from three biological replicates and three technical replicates, and the error bar is the standard deviation. ns indicates that there are no significant differences in gene expression, and asterisks indicate that there are significant differences in gene expression (Student's *t* test, *p < 0.05, ***p < 0.001).

To further understand the effect of MtUC1 on the functional genes involved in nodule development and symbiotic nitrogen fixation, RNA‐seq analysis was carried out on the 28‐dpi nodules of wild‐type R108 and *uc1* mutant. The results showed that compared to wild‐type R108, 244 genes were significantly downregulated and 369 genes were significantly upregulated in the 28‐dpi nodules of *uc1* mutant. GO enrichment analysis of differentially expressed genes in the nodules of the *uc1* mutant showed that most of the downregulated genes have cation‐binding functions, and some of them encode NCR peptides. The significantly upregulated genes were mainly enriched in the nucleus, and most of them possess transcriptional regulator activity (Figure 6a, Table S4). In addition, among the significantly upregulated genes in the nodules of *uc1* mutant, there were several genes related to senescence and defence responses, including CP, senescence regulatory factor and chitinase genes (Table S4). The significant upregulation of the CP genes was fully consistent with the previous correlation assay in the nodules of *uc1* mutant (Figure 3g), further demonstrating that mutation of *MtUC1* accelerated nodule senescence. In addition, RT‐qPCR results showed that 6 genes related to senescence and defence responses were significantly induced in *uc1* mutant nodules compared with wild‐type nodules (Figure 6b), while 11 genes encoding NCR peptides were significantly repressed (Figure 6c), which was consistent with the transcriptome sequencing results.

In addition, RT‐qPCR was performed to analyse several representative *MtNCR* genes (such as *MtNCR211*, *MtNCR169*, *MtNCR247* and *MtNCR343*) in inoculated roots at 7 dpi and 14 dpi. The results revealed a significant downregulation of these *MtNCR* genes in *uc1* mutant at both 7 dpi and 14 dpi relative to the wild‐type control (Figure S4).

### 2.4 MtUC1 Interacted With MtBI‐1 in Yeast Cells and *Nicotiana benthamiana* Leaf Cells

**FIGURE 7:**
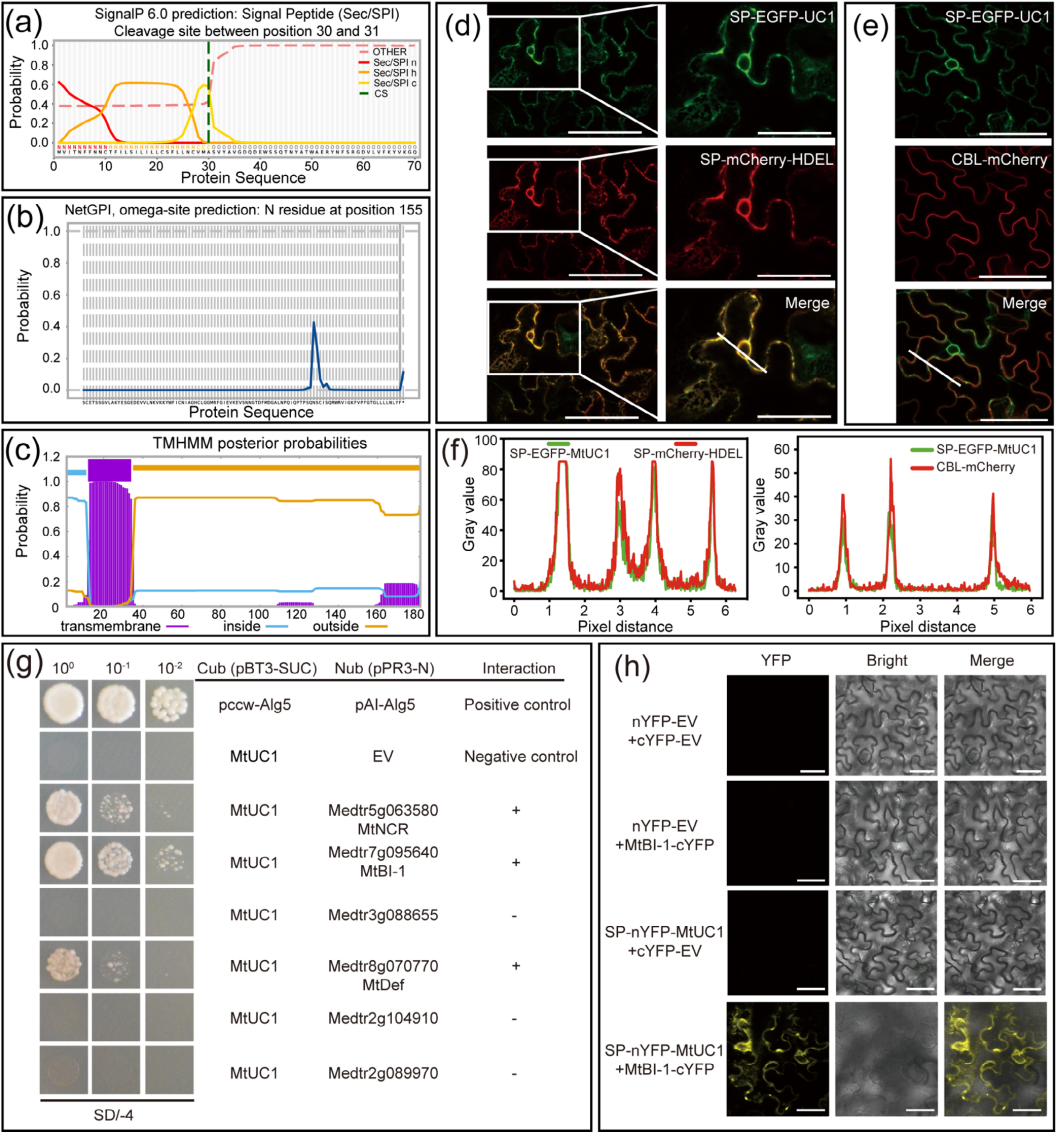
MtUC1 interacted with MtBI‐1 in yeast cells and *Nicotiana benthamiana* leaf cells. (a–c) Protein domain prediction for signal peptide (a), GPI anchor site (b) and transmembrane domain (c) of MtUC1. (d) SP‐EGFP‐MtUC1 (green) and SP‐mCherry‐HDEL (red) were co‐expressed in *N. benthamiana* leaf cells, and the merging between the green fluorescence signal and the red fluorescence signal was observed. The insets (right) are enlargements in the white boxes (left). Scale bars, 40 μm. (e) SP‐EGFP‐MtUC1 (green) and CBL‐mCherry (red) were co‐expressed in *N. benthamiana* leaf cells, and the merging between the green fluorescence signal and the red fluorescence signal was observed. Scale bars, 40 μm. (f) Intensity profiles of SP‐EGFP‐MtUC1 and SP‐mCherry‐HDEL (left) or CBL‐mCherry (right). Plots show fluorescence intensities of SP‐EGFP‐MtUC1 (green) and SP‐mCherry‐HDEL (red) or CBL‐mCherry (red) in the white line area (d, e). (g) Yeast point‐to‐point validation of the interaction between MtUC1 and candidate proteins. Pccw‐Alg5/pAl‐Alg5 and pBT3‐SUC‐MtUC1/pPR3‐N were used as positive and negative controls, respectively. (h) Bimolecular fluorescence complementation assay of the interaction between MtUC1 and MtBI‐1 in *N. benthamiana* leaf cells. nYFP, N‐terminal fragment of the yellow fluorescence protein; cYFP, C‐terminal fragment of the yellow fluorescence protein. Scale bars, 40 μm.

Based on the structure of the MtUC1 protein, it was predicted to have a signal peptide (SP), a glycosylphosphatidylinositol (GPI) anchor site and a transmembrane domain (Figure 7a–c), indicating that MtUC1 might be a secreted protein and might be localised on the outer surface of the cytoplasmic membrane. The subcellular localisation results of MtUC1 in *N. benthamiana* leaf cells showed that the green fluorescence signal of SP‐EGFP‐MtUC1 merged with the red fluorescence signal of SP‐mCherry‐HDEL (ER retention signal, His‐Asp‐Glu‐Leu) or CBL (Calcineurin B‐like)‐mCherry, indicating that MtUC1 was localised in the ER and plasma membrane (PM) (Figure 7d–f). Therefore, the *M. truncatula* cDNA membrane library was subsequently selected for Y2H experiments for the target protein.

Y2H screening and sequencing results showed that a total of 61 proteins that may interact with MtUC1 were identified (Table S5). The expression of these 61 genes was analysed in the different areas of nodules using the Symbimics database, and six genes with the same or similar expression pattern in nodules as *MtUC1* were selected as the candidate interacting proteins of MtUC1 for subsequent experiments (Figure S5).

The interactions between MtUC1 and the candidate interacting proteins were verified by yeast point‐to‐point experiments. The formation of yeast colonies showed that MtUC1 protein could bind with bax‐inhibitor 1 (Medtr7g095640, MtBI‐1), nodule‐specific cysteine‐rich peptide (Medtr5g063580, MtNCR), and defensin (Medtr8g070770, MtDef) (Figure 7g). MtBI‐1, which had relatively better yeast growth in the yeast point‐to‐point experiments, was selected for the BiFC experiment. The results showed that MtUC1 interacted with MtBI‐1 in the *N. benthamiana* leaf cells (Figure 7h).

**FIGURE 8:**
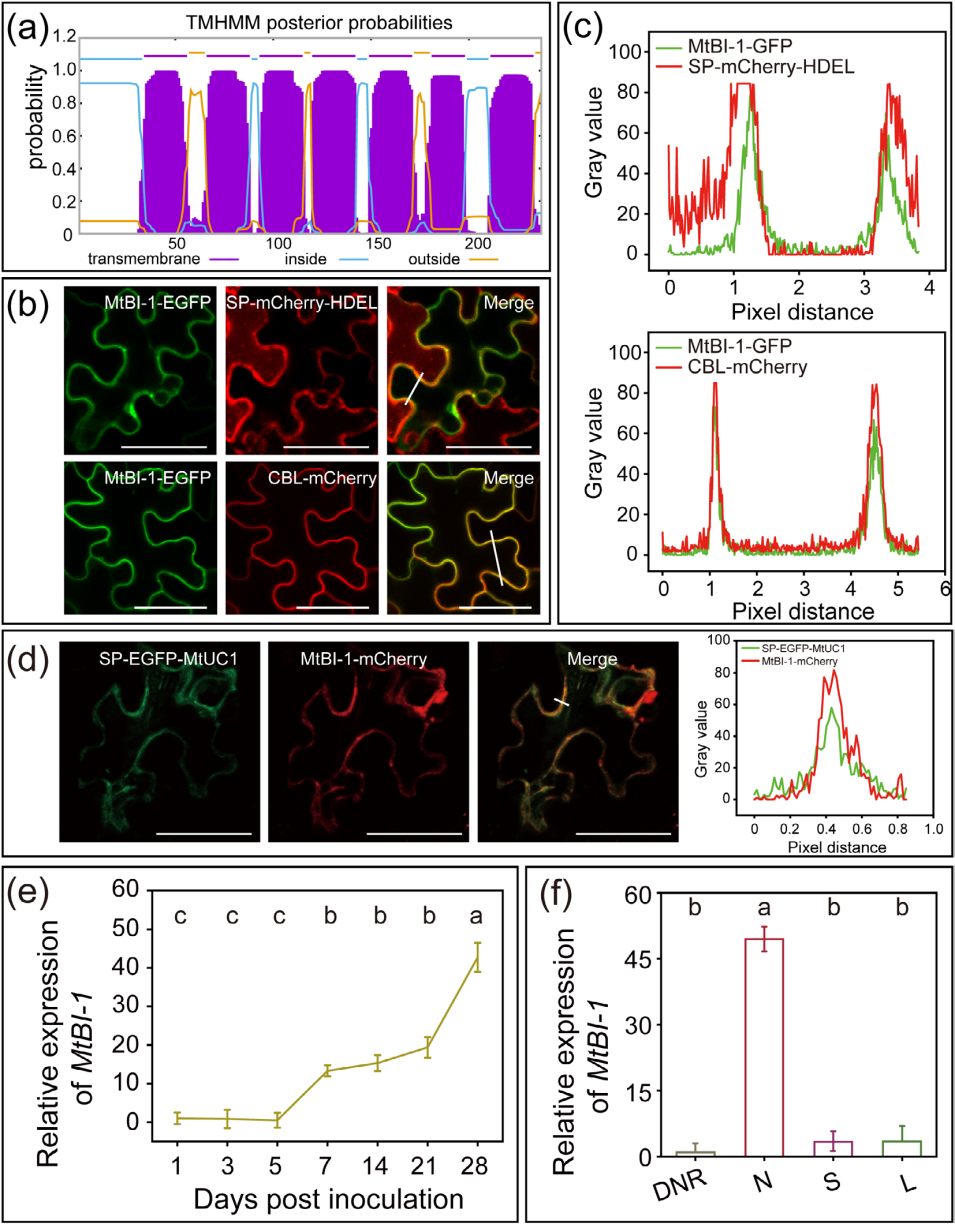
Expression pattern of *MtBI‐1*. (a) Prediction of the MtBI‐1 transmembrane domain. (b) Co‐expression of MtBI‐1‐EGFP (green) and SP‐mCherry‐HDEL (red, upper) or CBL‐mCherry (red, lower) in *Nicotiana benthamiana* leaf cells. The merging between the green fluorescence signal and the red fluorescence signal was observed. Scale bars, 40 μm. (c) Fluorescence intensity profiles of MtBI‐1‐EGFP and SP‐mCherry‐HDEL (upper) or CBL‐mCherry (lower). Plots show the fluorescence intensity of MtBI‐1‐EGFP (green) and SP‐mCherry‐HDEL (red) or CBL‐mCherry (red) in the white line area (b). (d) Co‐expression of SP‐EGFP‐MtUC1 (green) and MtBI‐1‐mCherry (red) in *N. benthamiana* leaf cells. The merging between the green fluorescence signal and the red fluorescence signal was observed. Scale bars, 40 μm. Plots show the fluorescence intensity of SP‐EGFP‐MtUC1 (green) and MtBI‐1‐mCherry (red) in the white line area (left). (e, f) Spatial and temporal expression patterns of *MtB‐1I*. Roots were obtained at 1, 3, 5, 7, 14, 21 and 28 days post‐inoculation (dpi) (e), and the denodulated roots (DNR), nodules (N), stems (S) and leaves (L) were harvested at 28 dpi (f). *MtActin* and *MtEF* were used as reference genes. Values show mean ± standard deviation for a total of three biological repeats, with three technical repeats included in each biological repeat. The same lowercase letters show no significant difference, and different lowercase letters show a significant difference (Tukey's test, *p* < 0.05).

The protein structure prediction results showed that MtBI‐1 has seven transmembrane domains (Figure 8a). The subcellular localisation results of MtBI‐1 in *N. benthamiana* leaves showed that green fluorescence‐labelled MtBI‐1‐EGFP co‐localised with red fluorescence‐labelled SP‐mCherry‐HDEL and CBL‐mCherry, indicating that MtBI‐1 was localised in the ER and PM (Figure 8b,c). In addition, the results of co‐localisation analysis showed that SP‐EGFP‐MtUC1 co‐localised with MtBI‐1‐mCherry in the leaf cells (Figure 8d).

### 2.5 *MtBI* *‐1* Was Highly Expressed in the 28‐dpi Nodules

The expression level assay showed that *MtBI‐1* was strongly induced mainly in the middle and late stages of nodule formation, especially in the 28 dpi inoculated roots (Figure 8e). Also, the *MBI‐1* transcript level was analysed in the denodulated roots, nodules, stems and leaves, and the results showed that *MtBI‐1* was expressed at the highest level in the root nodules (Figure 8f), similar to the expression pattern of *MtUC1* (Figure 1a,b).

## 3 Discussion

A few studies have shown that PC family members are involved in the symbiotic nitrogen fixation process between legumes and rhizobia, such as Ps/VsENOD5 (Scheres et al. 1990; Vijn et al. 1995), GmENOD55/GmN315 (de Blank et al. 1993; Kouchi and Hata 1993), MtENOD16 and MtENOD20 (Greene et al. 1998; Oztas 2017; Sun et al. 2019; Vernoud et al. 1999). However, the specific functions and mechanisms of most PC proteins in the nodule symbiosis remain unclear. In this study, *MtUC1*, a member of the PC family, was analysed to reveal its function in the nodule symbiosis. Results showed that *MtUC1* was mainly expressed in the interzone and vascular tissues of nodules. RNAi and mutation of *MtUC1* impaired the nitrogen‐fixing ability of nodules and accelerated the senescence of nodules. Additionally, MtUC1 interacted with MtBI‐1 in the ER and PM. In short, MtUC1 may prevent the premature senescence of nodules by interacting with MtBI‐1.

### 3.1 MtUC1 Maintains the Nodule Nitrogen Fixation Capacity

With the ageing of nodules, the activity of nitrogenase and the content of Lb decreased (Zhou et al. 2021), and the activity of protein hydrolases, especially CPs, increased (Pérez Guerra et al. 2010). CP gene families have been reported to be markers of the early stages of nodule senescence, such as GmCYSP1 (Alesandrini et al. 2003), AsNodf32 (Naito et al. 2000), PsCYP1 and PsCYP15A (Vincent and Brewin 2000). The reduction in Lb content (Figures 2a and 3e) and the increase in CP activity (Figures 2a and 3d) in *MtUC1‐RNAi* inoculated roots and *uc1* mutant nodules indicated that the absence of MtUC1 was associated with the senescence of root nodules.

In indeterminate nodules, early signs of senescence occur in some infected cells in the centre of the nitrogen‐fixing zone and gradually expand to the proximal cell layer in the periphery of the nodule, and finally the nodule central tissues collapse (Pérez Guerra et al. 2010). Ultrastructural analysis of the mature nodule cells of *M. truncatula* revealed that numerous vesicles, peroxisomes, ER, mitochondria and Golgi apparatus appeared in the infected cells of the senescent nodules, and that degradation of bacteroids and disintegration of symbiosome membranes resulted in the accumulation of damaged ‘ghost’ membranes in the cytoplasm, eventually leading to the death of the plant cells (Puppo et al. 2005; Van de Velde et al. 2006; Zhou et al. 2021). Both paraffin sections and ultrathin sections of *MtUC1‐RNAi* and *uc1* mutant nodules showed senescence‐related cytological changes (Figures 2b–g and 4). Therefore, RNAi or mutation of *MtUC1* caused the degeneration of bacteroids, reduced the symbiotic nitrogen‐fixing ability of nodules and accelerated the senescence of nodules.

### 3.2 MtUC1 Was Involved in the Survival of Bacteroids in the Infected Cells

Silencing and mutation of *MtUC1* led to reduced root nodule formation and degeneration of bacteroids within the nodule cells (Figures 2, 3c and 4). The RNA‐seq analysis showed that a large number of *NCRs* were downregulated in the 7‐dpi inoculated roots and the 28‐dpi nodules of *uc1* mutant (Figures 5e and 6c, Tables S2 and S4). NCR peptides have been identified to control the terminal differentiation of bacteroids and be required for an effective symbiosis on the inverted repeat‐lacking clade legumes and some Dalbergoid species (Czernic et al. 2015; Mergaert et al. 2006; Van de Velde et al. 2010). In addition, several *NCR* genes from *M. truncatula* have been shown to be involved in nodule development and symbiosis. For example, NCR211 was shown to play a key role in the survival and optimal functioning of differentiated rhizobia in host cells (Kim et al. 2015). NCR169 was expressed exclusively in the interzone and nitrogen fixation zone, and its loss led to the failure of bacteroid differentiation and the abolition of symbiotic nitrogen fixation (Horváth et al. 2015). In addition to influencing the translation and cell cycle of *Sinorhizobium meliloti* through interactions with many rhizobial proteins, NCR247 has also been found to promote the uptake of iron by rhizobia as a haem‐sequestering peptide, and the acquired iron is essential for nitrogenase activity (Farkas et al. 2014; Sankari et al. 2022). Recently, NCR343 and NCR‐new35 have been revealed to play an important role in ensuring the viability of differentiated bacteroids for successful symbiosis (Horváth et al. 2023; Zhang et al. 2023).

Moreover, the loss of *MtUC1* led to the downregulation of the expression of other nodule‐specific genes, including genes encoding nodule‐specific PLAT domain proteins, NIN, NodGRPs and nodulin (Figure 5d–f, Table S2). MtNIN, a core regulatory factor of rhizobial infection and nodule organogenesis, was also identified to play a key role in the development of symbiosomes and the suppression of defence and premature senescence in the later stage of nodulation (Liu et al. 2021). Nodule‐specific PLAT domain proteins were reported to be required for the complete differentiation of rhizobia into nitrogen‐fixing bacteroids and successful nodule formation (Alunni et al. 2007; Pislariu et al. 2019; Trujillo et al. 2019). Nodulin genes are host plant genes specifically induced during the symbiosis process and are essential for the formation and function maintenance of nodules. Among them, MtENOD16 and MtENOD20 were localised in the peribacteroid space and were required for rhizobial infection and nodule development (Oztas 2017). The multifunctional channel protein NOD26 was a major component of the symbiosome membrane and could facilitate the bidirectional transport of water, ammonia and other small‐molecule solutes (such as glycerol and formamide) (Frare et al. 2022; Masalkar et al. 2010). Taken together, these data suggested that MtUC1 may contribute to sustaining effective symbiosis by ensuring the survival of differentiated bacteroids.

### 3.3 MtUC1 May Prevent the Premature Aging of Root Nodules by Interacting With MtBI‐1

Bax‐Inhibitor 1 (BI‐1) is a cell death inhibitor conserved in all eukaryotes and regulates cell death in plants under abiotic stress and pathogen invasion. Studies in different plants have shown that the function loss of BI‐1 leads to severe programmed cell death phenotypes under abiotic stress, while overexpression of *BI‐1* attenuates programmed cell death caused by pathogen invasion (Babaeizad et al. 2009; Isbat et al. 2009; Ishikawa et al. 2009; Watanabe and Lam 2008, 2009). Also, BI‐1 was involved in the symbiosis between legumes and rhizobia. PvBI‐1a has been reported to play a dual role in the nodule symbiosis of *Phaseolus vulgaris* . *PvBI‐1a* was transiently induced after infection with rhizobia and promoted rhizobial infection by inhibiting the hypersensitive response in the early stage of symbiosis, while its overexpression led to the premature death of symbiotic nodule cells in the later stage of nodulation, thus affecting the nitrogen fixation efficiency (Hernández‐López et al. 2018). A transient increase in *BI‐1* expression after inoculation with rhizobia was also observed in soybeans (Brechenmacher et al. 2008). In this study, it was found that MtBI‐1 bound to MtUC1 (Figure 7g,h) and was strongly induced in the later stage of nodulation, especially in the 28‐dpi nodules (Figure 8e,f), which was similar to *MtUC1* (Figure 1a,b), suggesting that MtUC1 and MtBI‐1 mainly interact in the later nodule developmental stages. In addition, the function loss of *MtUC1* caused the upregulated expression of defence‐ and senescence‐related genes (Table S4). In *Phaseolus vulgaris* nodules, overexpression of *PvBI‐1a* induced the earlier expression of defence‐related genes and the premature death of nodule cells (Hernández‐López et al. 2018). Therefore, it is possible that MtUC1 may suppress the premature ageing of root nodules by interacting with MtBI‐1. However, the interaction mechanism between MtUC1 and MtBI‐1, as well as its precise biological function, remains to be further investigated.

## 4 Experimental Procedures

### 4.1 Plant Materials and Growth Conditions

*Medicago truncatula* wild‐type A17 and R108 were used in this study. Plants homozygous for a *Tnt1* transposon insertion in *MtUC1* were isolated from line NF10257 and designated as *uc1*. Genotyping primers were provided in Table S6.

Seeds were soaked in concentrated sulphuric acid for 8 min, washed with cold sterile water 6–8 times, sterilised with 30% sodium hypochlorite solution for 8 min, and then washed in sterile water 6–8 times. The sterilised seeds were incubated in sterile water for 3–5 h. The imbibed seeds were spread on water agar medium for 48 h at 4°C and germinated at 28°C. Germinated seedlings were transplanted into a sterilised vermiculite:perlite (volume ratio 2:1) mixture irrigated with nitrogen‐sufficient Fahräeus nutrient solution or nitrogen‐depleted Fahräeus nutrient solution. Plants were cultivated in a greenhouse under a 16‐h light/8‐h dark cycle at 24°C day/20°C night temperature conditions. For nitrogen‐deficient conditions, plants were inoculated with 5 mL of *S. meliloti* 1021 suspension (OD_600_ = 0.06) 7 days after the transfer. The same volume of sterile water was added to the roots for mock inoculation.

For spatiotemporal expression analysis of *MtUC1* and *MtB‐1I*, the inoculated roots at 1, 3, 5, 7, 14, 21 and 28 dpi, the denodulated roots, nodules, stems and leaves of the inoculated plants, and the uninoculated roots, stems and leaves at 28 dpi were harvested.

### 4.2 RNA Extraction and RT‐qPCR

Total RNA was extracted with the MiniBEST Plant RNA Extraction Kit (TaKaRa). Reverse transcription of mRNA was performed using HiScript II Q RT Supermix for qPCR (+gDNA wiper) (Vazyme). RT‐qPCR was conducted on the Applied Biosystems QuantStudio 6 Flex Real‐Time PCR System (Thermo Fisher Scientific) using ChamQ SYBR qPCR Master Mix (Vazyme). *MtActin* and *MtEF* were used as the internal controls for *M. truncatula* (Deng et al. 2019; Jiang et al. 2021), while *Sm16SrRNA* and *SmuppS* were used as internal controls for *S. meliloti* 1021. Gene expression was calculated using the 2^−ΔΔ*C*t^ method (Livak and Schmittgen 2001). The primers used in this experiment are listed in Table S6.

### 4.3 GUS Staining

The 2040 bp promoter fragment upstream of the *MtUC1* start codon was cloned into the pCAMBIA1391 vector carrying the *GUS* reporter gene. p*MtUC1*:GUS indicated in *A. rhizogenes* Ar.1193 were transformed into *M. truncatula* A17 roots as described previously (Wang, Yang, et al. 2023). Roots at 14 days (for sufficient nitrogen conditions) and inoculated roots at 7, 14 and 28 dpi were harvested, and histochemical GUS staining was performed as described previously (Wang, Yang, et al. 2023). Roots and nodules were observed in the bright field using a light microscope (BX53; Olympus). The experiments were repeated three times, and at least 10 transgenic roots were analysed each time.

### 4.4 RNAi in *M. truncatula* Transformed Hairy Roots

Two coding sequence fragments of *MtUC1* (220 bp and 217 bp) were cloned individually into the pK7GWIWG2D (II) vector to generate *MtUC1‐RNAi‐1* and *MtUC1‐RNAi‐2* (collectively referred to as *MtUC1‐RNAi*) constructs. The transformed *MtUC1‐RNAi* hairy roots were identified using green fluorescent protein (GFP) fluorescence under fluorescence microscopy (M165 FC; Leica). Inoculated roots were collected at 28 dpi for RNAi detection of *MtUC1*.

### 4.5 Nodulation Phenotype Analysis

Nodule numbers were counted at 28 dpi. CP activity was measured in *MtUC1‐RNAi* inoculated roots (28 dpi) and *uc1* mutant nodules (28 dpi) using the Plant Caspase ELISA Kit (Meimian). Lb content was assayed in *MtUC1‐RNAi* inoculated roots (28 dpi) and *uc1* mutant nodules (28 dpi) according to a published protocol (Jiang et al. 2021). In addition, transcript levels of *SmNif*s, *MtCP*s and *MtLB*s in *uc1* mutant nodules (28 dpi) were analysed. All assays were validated using three independent experiments.

For microscopic analysis, nodule sample fixation was performed with a 50% formaldehyde‐acetic acid‐alcohol solution. Following gradual dehydration of the sample using a series of increasing concentrations of ethanol gradients (30%, 50%, 70%, 85%, 95% and 100%), the sample was embedded in paraffin. Longitudinal sections (10 μm) were cut using a rotary microtome (RM2235; Leica) and stained with 0.05% toluidine blue prior to imaging with a light microscope. In addition, ultrathin sections were prepared to observe the internal morphology of the nitrogen‐fixing cells in the nodules. The nodules were fixed in 4% glutaraldehyde and embedded in resin. Double contrast staining was performed using uranyl acetate (2%) and lead citrate as dyes. Ultrathin sections (85 nm) were obtained using an ultrathin microtome (EMUC7; Leica) and examined using a transmission electron microscope (HT7700; Hitachi).

### 4.6 SYTO9‐Propidium Iodide Staining Analysis

To assess bacteroid viability in young nodules, live/dead cell staining was performed using the LIVE/DEAD BacLight Bacterial Viability Kit (Invitrogen) following the manufacturer's protocol. Nodules at 14 dpi were embedded in 3% (wt/vol) agarose and sectioned at 70 μm thickness using a VT 1000S vibratome (Leica). Sections were immediately incubated in the dark with a staining solution containing 5 μM SYTO9 and 30 μM propidium iodide (PI) for 2 min at room temperature. After staining, nodule sections were gently washed with sterile water to remove excess dye and mounted on glass slides. Fluorescence imaging was carried out using a confocal laser scanning microscope (TCS‐SP8 SR; Leica) with excitation/emission settings of 488/498–540 nm for SYTO 9 and 552/585–650 nm for PI. Images were analysed using Olympus CellSens software.

### 4.7 Subcellular Localisation

SP, GPI anchor site and transmembrane domain of MtUC1 and MtBI‐1 were predicted through the SignalP v. 6.0 online prediction website (https://services.healthtech.dtu.dk/service.php?SignalP) (Teufel et al. 2022), NetGPI‐1.1 online prediction website (https://services.healthtech.dtu.dk/service.php?NetGPI) (Gíslason et al. 2021) and TMHMM‐2.0 online prediction website (https://services.healthtech.dtu.dk/services/TMHMM‐2.0/) (Sonnhammer et al. 1998), respectively.

To construct subcellular localisation vectors for *N. benthamiana* leaves, SP‐EGFP‐MtUC1 and MtBI‐1‐EGFP were cloned into the pCAMBIA1300‐EGFP vector to generate *pMAS:SP‐EGFP‐MtUC1* and *pMAS:MtBI‐1‐EGFP* constructs, respectively. MtBI‐1‐mCherry was cloned into the p35S‐mCherry vector to generate the *p35S:MtBI‐1‐mCherry* construct.

CBL and HDEL are markers for PM and ER localisation, respectively. Therefore, CBL‐mCherry and SP‐HDEL‐mCherry were used as subcellular markers. SP‐EGFP‐MtUC1 or MtBI‐1‐EGFP were co‐expressed with CBL‐mCherry or SP‐HDEL‐mCherry in *N. benthamiana* leaves by infiltration. Transformed plants were grown in a greenhouse. After 3 days, the images were captured using a confocal laser scanning microscope (TCS‐SP8 SR; Leica). In addition, SP‐EGFP‐MtUC1 and MtBI‐1‐mCherry were co‐expressed in *N. benthamiana* leaves via infiltration.

All protein subcellular localisation assays were performed in at least three independent experiments. The filter sets for excitation (ex) and emission (em) were as follows: GFP (ex/em, 488 nm/498–540 nm) and mCherry (ex/em, 552 nm/585–650 nm). Images were analysed using Olympus CellSens software. Grey values were analysed using the ImageJ software.

### 4.8 Y2H Screen and Pairwise Interactions

The *MtUC1* coding sequence was cloned into four DUAL membrane bait vectors, pBT3‐N, pBT3‐C, pBT3‐SUC and pBT3‐STE, to obtain pBT3‐N‐MtUC1, pBT3‐C‐MtUC1, pBT3‐SUC‐MtUC1 and pBT3‐STE‐MtUC1, respectively. Coding sequences of *Medtr5g06358*, *Medtr7g095640 (MtBI‐1)*, *Medtr3g088655*, *Medtr8g070770*, *Medtr2g104910* and *Medtr2g089970* were cloned into the pPR3‐N vector to generate the corresponding pPR3‐N constructs.

The yeast strain NMY51 was transformed with the constructs in destination vectors using the lithium acetate‐mediated yeast transformation method according to the manufacturer's instructions (Yeast Protocols Handbook PT3024‐1; Clontech). The transformants were grown on minimal medium without the appropriate auxotrophic markers and in the presence of 3‐amino‐1,2,4‐triazole (3‐AT) at different concentrations. These assays were repeated three times.

### 4.9 BiFC Assays

SP‐nYFP‐MtUC1 was cloned into the pSPYNE‐35S vectors to generate the SP‐nYFP‐MtUC1 construct, and the *MtBI‐1* coding sequence was cloned into pSPYCE‐35S to generate the MtBI‐1‐cYFP construct. SP‐nYFP‐MtUC1 and MtBI‐1‐cYFP were co‐expressed in *N. benthamiana* leaves, and the transformed plants were grown in a greenhouse. After 3 days, the images were observed using a confocal laser scanning microscope. The filter sets for excitation and emission were as follows: YFP (ex/em, 514 nm/524–570 nm). The BiFC experiments were repeated three times, and at least five leaves were analysed each time.

### 4.10 Digital Expression Analysis of *MtPC*s Based on Microarray Analysis

The expression profiles of *MtPC*s were obtained from the Symbimics database (https://iant.toulouse.inra.fr/symbimics) (Roux et al. 2014). The expression patterns of *MtPC*s in the interzone of *M. truncatula* nodules were visualised using the pheatmap function implemented in R.

### 4.11 RNA‐Seq Analysis

Inoculated roots (7 dpi) and nodules (28 dpi) from wild‐type R108 and *uc1* mutants were sampled for RNA‐seq analysis. Three biological replicates were set up for each sample. The RNA‐seq data are available in the Beijing Institute of Genomics (BIG) under Bioproject PRJCA031356. Differential expression of genes in the same tissues was compared between wild‐type R108 and *uc1* mutants. The Benjamini–Hochberg (BH) procedure was used to correct for multiple hypothesis testing to control the false discovery rate (FDR), and genes with log_2_ fold change ≥ |2| and corrected *p*‐value (*p*_adj_) < 0.05 were considered significantly differentially expressed. GO (gene ontology) enrichment analysis of differentially expressed genes was performed using GOseq software (Young et al. 2010).

### 4.12 Statistical Analyses

Statistical significance was calculated using a two‐tailed Student's *t* test (*p < 0.05, **p < 0.01, ***p < 0.001), and error bars indicate the standard deviation (SD). A one‐way ANOVA followed by Tukey's multiple comparison test was used to determine differences. Statistical significance was set at α = 0.05. Statistical analyses were performed using IBM SPSS Statistics v. 25.0 and Origin software.

## Author Contributions

Li Wang was involved in investigation, formal analysis and writing – original draft preparation. Mengdi Zhang, Wenjun Tan, Zhaoyang Yang, Shicheng Zhao and Mengzhen Jia were involved in investigation. Gehong Wei was involved in resources. Minxia Chou was involved in conceptualisation and writing – review and editing.

## Conflicts of Interest

The authors declare no conflicts of interest.

## Supporting information


**Figure S1:** Relative read distribution of *MtPC*s in the different zones of *Medicago truncatula* nodules. Heat map of 50 *MtPC*s microarray expression data. IZ, interzone; ZI, meristem zone; ZIId, distal infection zone; ZIII, nitrogen fixation zone; ZIIp, proximal infection zone. Black shows low expression, and yellow shows high expression.

**Figure S2:** Two RNAi interference fragments of *MtUC1*. cDNA sequence alignment of MtUCs. Red lines indicate *MtUC1‐RNAi‐1* and *MtUC1‐RNAi‐2*.

**Figure S3:** Identification of *MtUC1‐RNAi* positive plants. Stereoscopic fluorescence microscope images of hairy roots of tissue culture seedlings (upper) and transformed plants (lower, 28 days post‐inoculation). Non‐fluorescent roots lacking the EGFP signal represent non‐transformed roots. Images are representative of the RNA interference positive plant identification experiment. Scale bar, 2.5 mm.

**Figure S4:** Relative expression levels of *MtNCR*s. Relative expression levels of *MtNCR211*, *MtNCR169*, *MtNCR247* and *MtNCR343* in wild‐type R108 and *uc1* mutant inoculated roots (7 and 14 days post‐inocuation [dpi]). *MtActin* and *MtEF* were used as reference genes. Values are derived from three biological replicates and three technical replicates, and the error bar is the standard deviation. ns indicates that there are no significant differences in gene expression (Student's *t*‐test).

**Figure S5:** Screening of candidate proteins interacting with MtUC1. The relative read distributions of *MtUC1* and six candidate interacting genes in different zones of *Medicago truncatula* nodules. Data were obtained from the Symbimics database. FI, meristem zone; IZ, interzone; FIId, distal infection zone; FIIp, proximal infection zone; ZIII, nitrogen fixation zone.

**Table S1:** Relative read distribution of *MtPC*s in the different zones of *Medicago truncatula* nodules.

**Table S2:** Significantly downregulated genes in the inoculated roots of *uc1* mutant (7 days post‐inoculation).

**Table S3:** Significantly upregulated genes in the inoculated roots of *uc1* mutant (7 days post‐inoculation).

**Table S4:** Significantly differentially expressed genes in the nodules of *uc1* mutant (28 days post‐inoculation).

**Table S5:** Proteins screened by yeast two‐hybrid assay that may interact with MtUC1.

**Table S6:** Primers used in this study.

## Data Availability

The raw sequence data is accessible at the Genome Sequence Archive in BIG (Beijing Institute of Genomics) Data Center with accession number PRJCA031356.
