# Supplementary material for: Uclacyanin MtUC1 Is Involved in the Regulation of Nodule Senescence in Medicago truncatula
Source: Mol Plant Pathol. 2025 Nov 12;26(11):e70171. doi: 10.1111/mpp.70171 (PMC12612560; doi:10.1111/mpp.70171)
Supplement: Supplementary file 1 — Figure S1: Relative read distribution of MtPCs in the different zones of Medicago truncatula nodules. Heat map of 50 MtPCs microarray expression data. IZ, interzone; ZI, meristem zone; ZIId, distal infection zone; ZIII, nitrogen fixation zone; ZIIp, proximal infection zone. Black shows low expression, and yellow shows high expression. [file MPP-26-e70171-s008.docx]

**Figure S1 Relative read distribution of *MtPC*s in the different zones of *M. truncatula* nodules.**


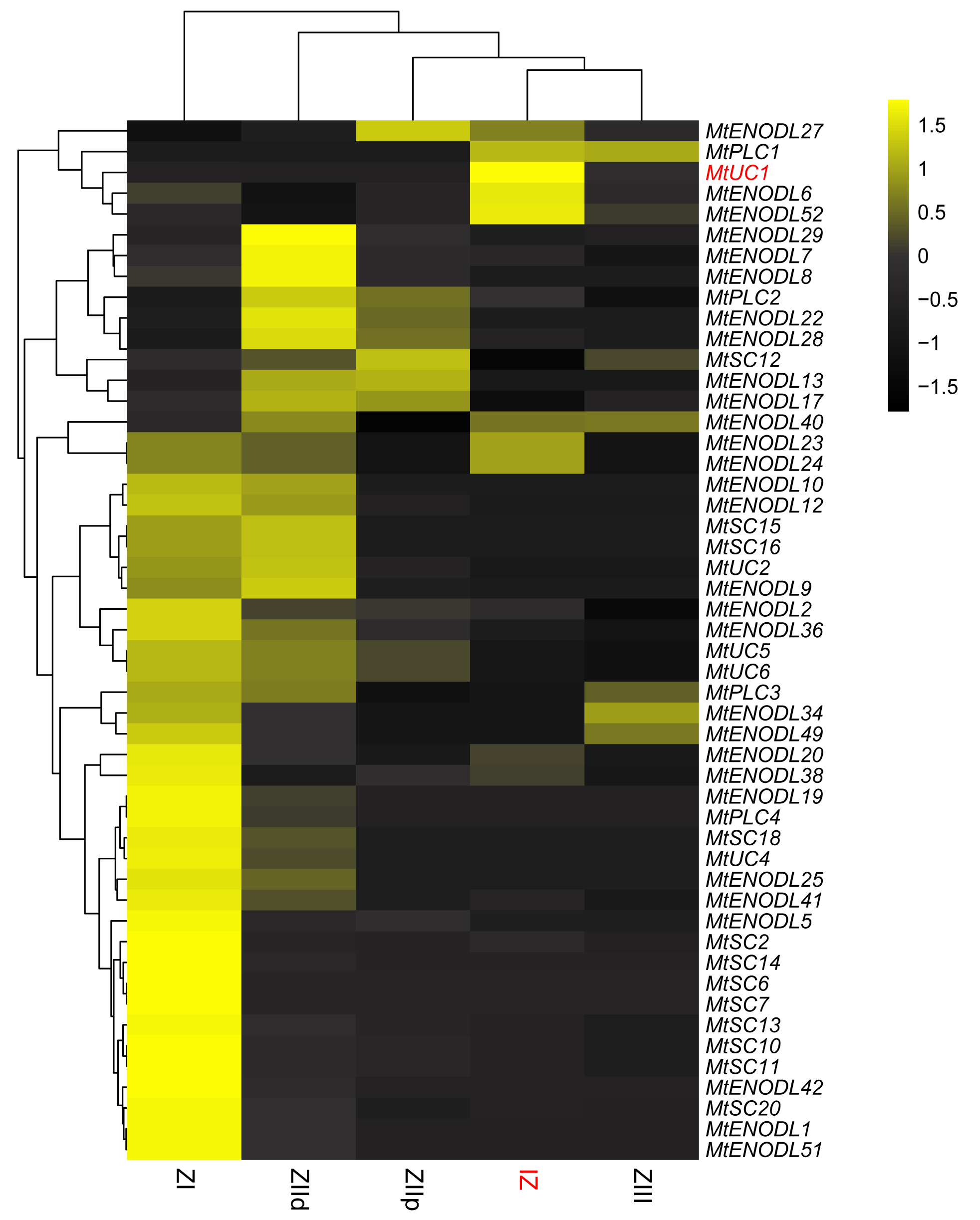


Heat map of 50 *MtPC*s microarray expression data. ZI, meristem zone; ZIId, distal infection zone; ZIIp, proximal infection zone; IZ, interzone; ZIII, nitrogen fixation zone. Black shows low expression, and yellow shows high expression.
