# Supplementary material for: Uclacyanin MtUC1 Is Involved in the Regulation of Nodule Senescence in Medicago truncatula
Source: Mol Plant Pathol. 2025 Nov 12;26(11):e70171. doi: 10.1111/mpp.70171 (PMC12612560; doi:10.1111/mpp.70171)
Supplement: Supplementary file 2 — Figure S2: Two RNAi interference fragments of MtUC1. cDNA sequence alignment of MtUCs. Red lines indicate MtUC1‐RNAi‐1 and MtUC1‐RNAi‐2. [file MPP-26-e70171-s006.docx]

**Figure S2 Two RNAi interference fragments of *MtUC1*.**


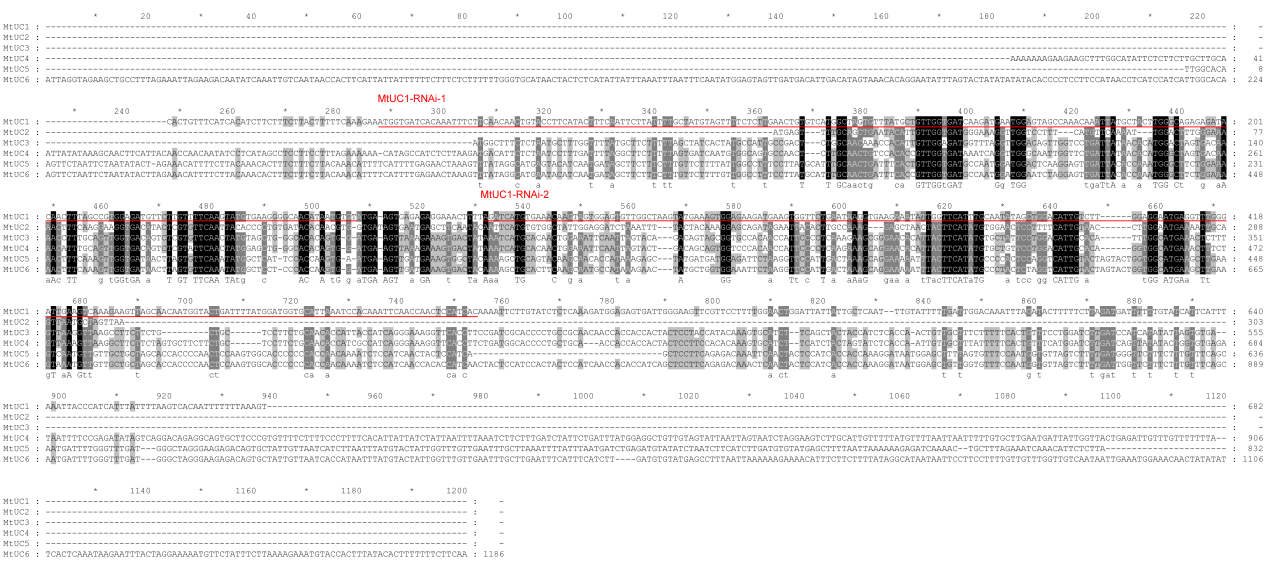


cDNA sequence alignment of MtUCs. Red lines indicate *MtUC1-RNAi-1* and *MtUC1-RNAi-2*
