# Supplementary material for: Uclacyanin MtUC1 Is Involved in the Regulation of Nodule Senescence in Medicago truncatula
Source: Mol Plant Pathol. 2025 Nov 12;26(11):e70171. doi: 10.1111/mpp.70171 (PMC12612560; doi:10.1111/mpp.70171)
Supplement: Supplementary file 3 — Figure S3: Identification of MtUC1‐RNAi positive plants. Stereoscopic fluorescence microscope images of hairy roots of tissue culture seedlings (upper) and transformed plants (lower, 28 days post‐inoculation). Non‐fluorescent roots lacking the EGFP signal represent non‐transformed roots. Images are representative of the RNA interference positive plant identification experiment. Scale bar, 2.5 mm. [file MPP-26-e70171-s002.docx]

**Figure S3 Identification of *MtUC1-RNAi* positive plants.**

**
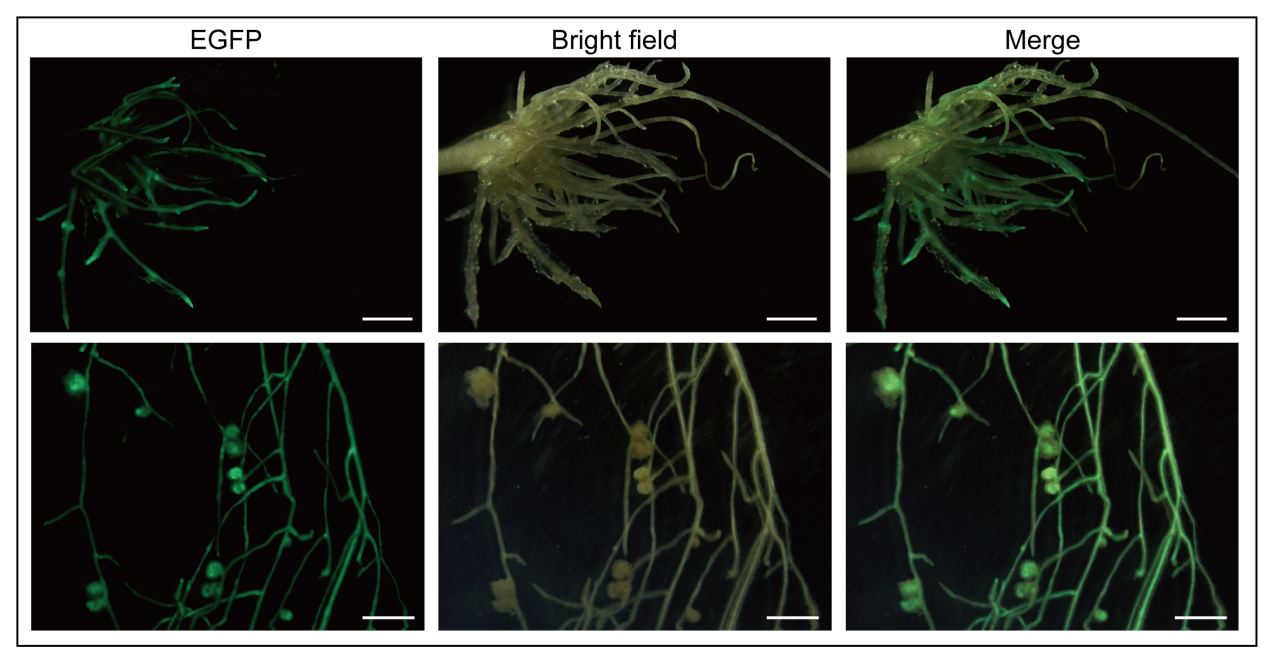
**

Stereoscopic fluorescence microscope images of hairy roots of tissue culture seedlings (upper) and transformed plants (lower, 28 dpi). Non-fluorescent roots lacking the EGFP signal represent non-transformed roots. Images are representative of the RNA interference positive plant identification experiment. Scale bar, 2.5 mm.
