# Supplementary material for: Uclacyanin MtUC1 Is Involved in the Regulation of Nodule Senescence in Medicago truncatula
Source: Mol Plant Pathol. 2025 Nov 12;26(11):e70171. doi: 10.1111/mpp.70171 (PMC12612560; doi:10.1111/mpp.70171)
Supplement: Supplementary file 4 — Figure S4: Relative expression levels of MtNCRs. Relative expression levels of MtNCR211, MtNCR169, MtNCR247 and MtNCR343 in wild‐type R108 and uc1 mutant inoculated roots (7 and 14 days post‐inocuation [dpi]). MtActin and MtEF were used as reference genes. Values are derived from three biological replicates and three technical replicates, and the error bar is the standard deviation. ns indicates that there are no significant differences in gene expression (Student's t‐test). [file MPP-26-e70171-s007.docx]

**Figure S4 Relative expression levels of *MtNCR*s.**


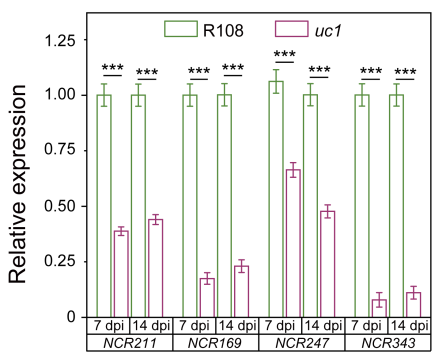


Relative expression levels of *MtNCR211*, *MtNCR169*, *MtNCR247*, and *MtNCR343* in wild-type R108 and *uc1* mutant inoculated roots (7 dpi and 14 dpi). *MtActin* and *MtEF* were used as reference genes. Values are derived from three biological replicates and three technical replicates, and the error bar is the standard deviation. ns indicates that there are no significant differences in gene expression (Student's t-test).
