# Supplementary material for: Uclacyanin MtUC1 Is Involved in the Regulation of Nodule Senescence in Medicago truncatula
Source: Mol Plant Pathol. 2025 Nov 12;26(11):e70171. doi: 10.1111/mpp.70171 (PMC12612560; doi:10.1111/mpp.70171)
Supplement: Supplementary file 5 — Figure S5: Screening of candidate proteins interacting with MtUC1. The relative read distributions of MtUC1 and six candidate interacting genes in different zones of Medicago truncatula nodules. Data were obtained from the Symbimics database. FI, meristem zone; IZ, interzone; FIId, distal infection zone; FIIp, proximal infection zone; ZIII, nitrogen fixation zone. [file MPP-26-e70171-s010.docx]

**Figure S5 Screening of candidate proteins interacting with MtUC1.**

**
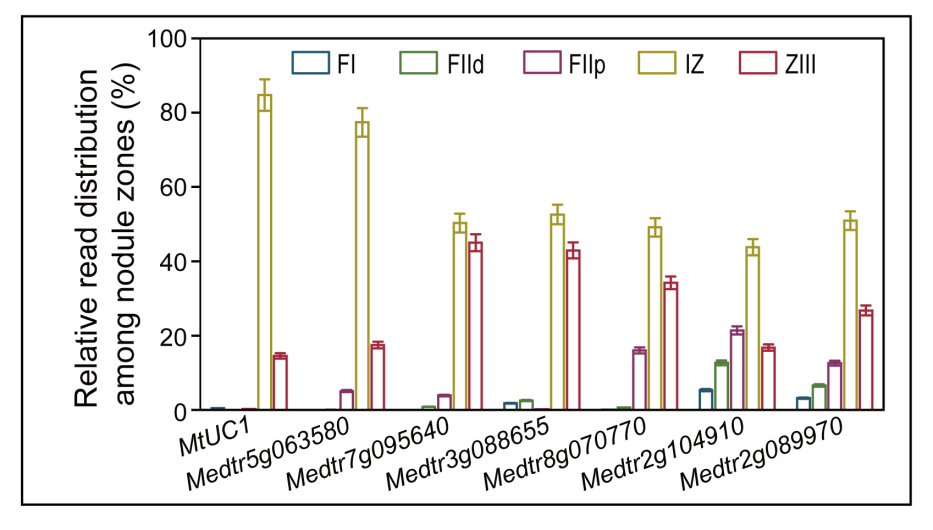
**

The relative read distributions of *MtUC1* and six candidate interacting genes in different zones of *M. truncatula* nodules. Data were obtained from the Symbimics database. FI, meristem zone; FIId, distal infection zone; FIIp, proximal infection zone; IZ, interzone; ZIII, nitrogen fixation zone.
